# Accelerating an Olympic Decathlete’s Return to Competition Using High-Frequency Blood Flow Restriction Training: A Case Report

**DOI:** 10.3390/sports13120436

**Published:** 2025-12-04

**Authors:** Chris Gaviglio, Stephen P. Bird

**Affiliations:** School of Health and Medical Sciences, University of Southern Queensland, Ipswich 4305, Australia; stephen.bird@unisq.edu.au

**Keywords:** blood flow restriction, rehabilitation, muscular injury, track and field

## Abstract

This case report describes the acceleration of an Olympic decathlete’s return to competition induced via high-frequency Blood Flow Restriction (BFR) training. BFR has gained popularity as an innovative rehabilitation method for promoting muscle repair and adaptation through anabolic and regenerative pathways when high mechanical loading is not possible. A 26-year-old elite decathlete with nine years of international experience sustained a Grade 2b strain of the semimembranosus and semitendinosus (a 9 mm central tendon tear) during a hurdle sprint. The injury was confirmed via MRI two days post-injury. Grade 2b hamstring injuries with intramuscular tendon involvement commonly require up to 4 weeks of rehabilitation before full training can be resumed. With the athlete due to complete in an Olympic Games competition 17 days post-injury, an intensive BFR-assisted rehabilitation program was initiated. Over 12 consecutive days, the athlete completed 3–6 BFR sessions per day (20–30 min each) at 50% limb occlusion pressure, along with physiotherapy and pain-limited functional testing. BFR was applied passively for recovery, during conditioning, and in low-load strength sessions. By day 12, sprint velocity reached 95% maximum, and the athlete successfully completed the decathlon, with no adverse effects or reinjury. This case illustrates how high-frequency BFR-assisted rehabilitation may facilitate accelerated recovery from a hamstring injury, enabling an effective return to elite competition within condensed timelines.

## 1. Introduction

Hamstring strains constitute the most prevalent injury in elite track and field, accounting for up to 37% of all muscle injuries in this population [1]. The biceps femoris is most frequently involved, followed by the semimembranosus and semitendinosus muscles [2]. Muscle injury classification systems provide a framework for muscle injury diagnosis [3]. To standardise classification and prognosis, the British Athletics Muscle Injury Classification provides a magnetic resonance image (MRI)-based classification system with clearly defined, anatomically focused classes based on the site and severity of the injury, graded from 0 to 4 [4]. Higher-grade and intratendinous (‘c’) injuries are associated with a longer time until one can return to full training (RTFT) and a higher risk of recurrence [3]. Median RTFTs range from approximately 18 days for Grade 1a injuries to 84 days for Grade 3c injuries. Within this framework, Grade 2b muscle–tendon junction injuries typically require 14–28 days (mean 18.6 days) of healing until one can return to full training [3].

Traditional rehabilitation protocols emphasise progressive mechanical loading, including eccentric strengthening, neuromuscular control, and graded running exposure [3,5]. However, despite well-established rehabilitation frameworks, elite athletes preparing for major competitions often face compressed timelines that challenge traditional loading progression. In these contexts, interventions that accelerate strength restoration and neuromuscular recovery without increasing reinjury risk are of high practical value. One such method is Blood Flow Restriction (BFR) training, which combines low-load exercise with partial vascular occlusion to mimic the responses of high-intensity training [6,7]. BFR involves the application of external pressure to the proximal portion of the upper thigh or arm during exercise. External pressure is applied, corresponding to 40–80% of the individual’s limb occlusion pressure (LOP) [8,9]. This controlled restriction reduces venous return whilst maintaining partial arterial inflow, creating a localised hypoxic environment with a subsequent increase in metabolic stress within the muscle. The resulting accumulation of metabolites and increased recruitment of higher-threshold motor units activate anabolic pathways, promoting muscle protein synthesis and metabolic adaptations [6,10,11], making BFR valuable when high mechanical loads are contraindicated due to injury. Additionally, BFR has been shown to mitigate muscle atrophy [12,13], and promote tendon remodelling [14,15] during periods of limited loading. BFR can also be applied passively through a process called ischemic preconditioning (IPC), which consists of repeated short cycles of limb ischemia followed by reperfusion [16,17]. IPC acutely increases muscle reoxygenation and repeated-force capacity and, when applied repeatedly or before high-intensity training, has been shown to improve time-trial and muscular power outputs while reducing fatigue indices in athletes [18,19,20,21,22].

The decathlon is one of the most physiologically demanding sports in track and field, combining ten disciplines (including sprints, hurdles, jumps, throws, and endurance) and being completed over two consecutive days [23]. Injury incidence rates during elite decathlon competitions have been reported to be 210 injuries per 1000 athlete starts, with half of all injures classified as traumatic (50%) and most affecting the muscle (30.3%) or tendon (21.2%) and occurring predominately at the thigh (22.7%) [24]. Moreover, in the cited instance, 3.5% of athletes did not start on Day 2, and 22.3% failed to complete the decathlon, underscoring the event’s high physical toll and risk of injury. This case report describes the accelerated rehabilitation of an elite decathlete who sustained a BAMIC Grade 2b semimembranosus hamstring injury with intramuscular tendon involvement. In this rehabilitation intervention, we employed a high-frequency, multi-modal BFR protocol that integrated resistance, aerobic, and passive modalities to optimise recovery and preserve performance readiness. Specifically, this study outlines the program design, daily progression, and outcomes, along with potential theoretical mechanisms that may have facilitated the athlete’s successful return to full competition within a condensed timeframe.

## 2. Materials and Methods

### 2.1. Case Presentation

**Figure 1:**
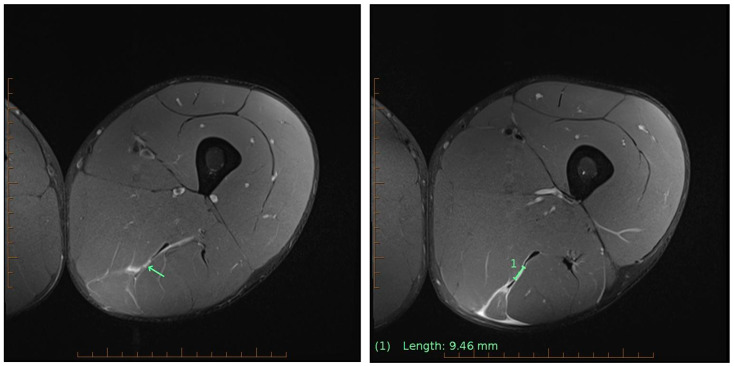
Post-injury magnetic resonance imaging classified the affliction as a BAMIC Grade 2b hamstring injury. Green arrows indicate key prognostic features.

The athlete was a 26-year-old elite male decathlete with nine years of international competition experience. During a hurdle sprint race at a pre-Olympic preparation camp (2021 Tokyo Olympic Games), the athlete experienced sharp pain in his left posterior thigh and immediately ceased running. MRI was performed using a 3T scanner 48 h post-injury and revealed a Grade 2b injury involving the semimembranosus and semitendinosus, with a 9.46 mm central tendon tear of the semimembranosus. No free-tendon discontinuity or retraction was observed (Figure 1). According to the British Athletics Muscle Injury Classification (BAMIC), a Grade 2b injury typically corresponds to a four-week return-to-training timeline under conventional rehabilitation protocols [3].

Given the proximity to the corresponding Olympic Games competition (17 days post-injury), conventional rehabilitation timelines were incompatible with competition participation. Following consultation with the athlete’s coaching staff and medical personnel, an intensive evidence-based rehabilitation strategy incorporating high-frequency BFR training was adopted. All procedures were conducted under direct supervision and followed established guidelines (Australian Institute of Sport, 2021) for BFR application in athletic populations [1,2]. Due to the location of the training camp, condensed timelines, and imminent requirement to travel to the competition, objective strength testing or repeat imaging reassessments were not feasible. Accordingly, progressions were guided by functional tolerance, and daily clinical evaluations were carried out by the medical team. The athlete provided informed consent, and all procedures were performed in accordance with the ethical standards of the Declaration of Helsinki and approved by the Human Research Ethics Committee of the University of Southern Queensland (ETH2023-0591).

### 2.2. Rehabilitation Framework

The rehabilitation framework was designed by the lead strength-and-conditioning coach and refined in consultations with the treating physiotherapist and the athlete’s head coach. Physiotherapy was delivered concurrently and consisted of daily clinical assessments and conventional soft-tissues techniques employed to monitor symptoms, guide load tolerance, and support functional readiness. While each BFR modality employed (e.g., IPC and low-intensity BFR exercise) is supported by existing evidence [15,25,26,27,28], their integration into a single multimodal high-frequency program is yet to be studied and therefore represents an applied approach.

It was hypothesised that the acute responses would be additive, thereby promoting recovery and amplifying the physiological adaptations necessary for elite-level competition. BFR was applied using nylon pneumatic cuffs (TheBFR.co, Brisbane, Australia). The cuffs were positioned at the most proximal portion of each limb, immediately inferior to the inguinal crease for the thighs and just distal to the deltoid insertion for the upper arms. Lower-limb cuffs (10 cm width) were inflated to 50% limb occlusion pressure (LOP; 140 mmHg), and upper-limb cuffs (5 cm width) were inflated to 80% LOP (120 mmHg). LOP was calculated based on limb circumference and resting blood pressure, and inflation pressure was adjusted according to the width of the cuff [9,29]. Training intensity and exercise selection in relation to the injured hamstring were guided by symptom-limited tolerance (allowing low, non-sharp discomfort, ≤2/10 on the Borg CR-10) and daily functional assessment rather than pre-set load prescriptions [30].

### 2.3. Rehabilitation Program Overview

**Figure 2:**
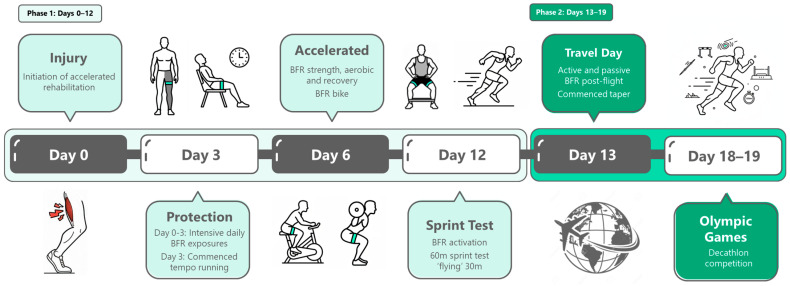
Schematic of the rehabilitation timeline.

An accelerated rehabilitation program was designed in two phases based on clinical and functional objectives and logistical constraints. Phase 1 (days 1–12) was an intensive period that incorporated the majority of daily BFR exposure, with two to six sessions per day, integrating muscle activation, strength, aerobic, and recovery sessions. Phase 2 (days 13–19) encompassed international travel, tapering, and competition (Figure 2). Early sessions in phase 1 prioritised upper-body BFR using banded resistance for muscular and systemic activation, right-leg high-load BFR strength training to provide a contralateral stimulus, and passive BFR application (ischemic preconditioning—IPC) to assist recovery. As tolerance improved, progressive hamstring-specific exercises and sport-specific gym-based exercises were introduced. Running commenced on Day 3 (5 × 30 m) and was increased in volume and intensity. On Day 12, the athlete was required to complete a 60 m sprint. The time to complete a 30 m sprint was recorded from the 30 m to 60 m mark (“flying” 30 m) at ≥ 95% based on his most recent maximal velocity. Successful completion served as the official medical and performance clearance for Olympic participation. In phase 2, the taper replicated the athlete’s usual pre-competition schedule with reduced training volume and less frequent BFR exposure. IPC sessions were maintained to support recovery and physiological readiness. On days 18 and 19, the athlete subsequently competed in the Olympic decathlon. Table 1 provides descriptions of the rehabilitation modalities, while Table 2 presents a summary of the accelerated rehabilitation program.

## 3. Results

The athlete completed 17 consecutive days of rehabilitation without experiencing any adverse events or reporting any setbacks. Daily programming was adapted according to tolerance, with progression determined by pain-free movement and functional performance. Pain exacerbation was minimal, with limited mechanical restrictions observed throughout the process. Due to the location of the pre-departure training camp and time constraints, standardised clinical strength assessment tools (e.g., dynamometry or force plates) were unavailable, and maximal strength testing was avoided to prevent fatigue during the pre-competition taper. As such, two field-based strength assessments were utilised. (1) Single-Leg Hamstring Isometric Push Test: In this test, the athlete lay supine on the ground with his foot and a digital bathroom scale elevated on a 20 cm box. The athlete was instructed to ‘push down’ onto the scale as hard as possible without lifting his hips off the ground or inducing hamstring pain. Measurements were taken in three different foot positions (neutral–dorsum facing superiorly; 30° medial rotation; and 30° lateral rotation). (2) Single-Leg Eccentric Hamstring Lower: This test was performed on a prone hamstring curl machine with a 3 sec eccentric action per repetition. The uninjured leg was trained in alternating sets, with a 3 min rest between sets. Both legs were used in the concentric action to return the weight to the starting point. These methods provided practical and repeatable indicators of relative limb strength and inter-limb symmetry. Improvements were observed in the injured (left) limb between days 7 and 12 (Table 3). By Day 12, bilateral symmetry was achieved, corresponding with the athlete’s self-reported confidence in his hamstring’s ability to generate force and tolerate sprint-specific loading. The SL Hamstring Isometric Push Test was performed daily as a readiness marker to compare maximal downward force between limbs (data unavailable).

### 3.1. Running Progression

Running was introduced on day 3, with the initial sessions emphasising an upright posture and symmetrical ground contact to reinforce normal gait mechanics. Progressive improvements in sprint performance were evident from day 6 to day 12, as presented in Table 4. To obtain medical clearance for Olympic competition, the athlete was required to complete a flying 30 m sprint in ≤ 3.04 s by day 12, corresponding to 95% of his pre-injury maximal sprint velocity derived from race performance. By day 12, the athlete exceeded this criterion, achieving 2.99 sec for the flying 30 m effort without pain or alternate running mechanics.

### 3.2. Return to Competition

After receiving medical clearance on day 12, the athlete travelled with the national team to the Olympic Games. Training during this period was limited to light activation, IPC, and event-specific sessions consistent with his usual pre-competition taper. He successfully completed all ten decathlon events without recurrence of symptoms or loss of function. Comparative performance data are summarised in Table 5, providing the Olympic results relative to the athlete’s decathlon personal best (PB) achieved six months prior. Performance in several disciplines was influenced by contextual factors: the athlete adopted a cautious approach in the 110 m hurdles due to the previous injury mechanism; his javelin performance was affected by the high braking forces placed on the front (left) block leg [31]; and the slower 1500 m time resulted from a deliberate pacing strategy adopted to support a teammate, as he was no longer in medal contention after recording a no-height in the pole vault. No post-competition imaging was available due to international travel restrictions and mandatory quarantine. The athlete reported no pain, swelling, or functional limitation during or following the competition and resumed full training post-quarantine without re-injury.

## 4. Discussion

This case report details the integration of an accelerated high-frequency BFR rehabilitation program that enabled an elite decathlete to return to an Olympic Games competition after suffering a Grade 2b semimembranosus and semitendinosus strain with central tendon involvement. Contrary to our findings, the literature suggests that conventional timelines for returning to full training for comparable injuries range from 4 to 6 weeks [3,32]. The rehabilitation framework used in this case report integrated high-frequency BFR in active and passive modalities to promote healing and recovery and maintain physiological capacities and performance when traditional high-intensity loading was contraindicated. The program was informed by evidence supporting the role of BFR as a physiological catalyst for muscle repair and regeneration and the maintenance of strength characteristics. While biochemical markers were not assessed in this case, low-intensity BFR exercise is reported to provoke immune and myogenic responses characterised by macrophage and neutrophil recruitment, growth factors, and cytokine signalling (e.g., interleukin-6 [IL-6] and vascular endothelial growth factor [VEGF]) directing local satellite cells to the site of muscle damage to collectively facilitate tissue regeneration and remodelling [27,33,34]. Understanding the coordination between these cellular processes and the timing of mechanical loading is central to successful muscle rehabilitation [3,35].

BFR training promotes muscle hypertrophy and strength gains under low-load conditions, mediated through the mTOR, testosterone, growth hormone, and IGF-1 signalling pathways [10]. In this application, our primary goal was to preserve muscle cross-sectional area and contractile strength when mechanical loading was restricted. Lower-limb immobilisation can reduce muscle strength by 12–13% and muscle volume by 3.5–7% within one week [36,37,38]. Although muscle girth was not recorded, the athlete’s eccentric hamstring strength achieved parity by day 12 (Table 3), and he was able to tolerate lower-body strength exercises at pre-injury levels (data not presented). This suggests that the BFR program effectively attenuated strength loss and promoted rapid recovery of muscle function.

The importance of eccentric hamstring strength in sprint performance and rehabilitation is well established [3,39,40]. The single-leg eccentric hamstring lower served as both a strengthening exercise and an objective recovery marker, enabling timely rehabilitation assessment. The rapid restoration of sprint performance further suggests BFR may have contributed to the recovery of neuromuscular function. Injury and immobilisation result in neuromuscular inhibition, affecting maximal voluntary contraction ability and extending performance recovery time [41,42]. While speculative in this context, fast-twitch motor unit activation during the low-intensity BFR exercises [43,44] may have helped preserve or restore neural drive and inter-muscular coordination required for sprinting and event-specific performance.

Ischemic preconditioning (IPC) was implemented throughout as a passive BFR recovery modality, offering multiple complementary benefits. Repeated IPC protocols improve muscle oxidative recovery kinetics, reduce resting muscle oxygen consumption, and enhance mitochondrial function and vascular adaptation in skeletal muscle [17,26,45]. IPC acutely increases muscle perfusion and reoxygenation, and when applied regularly or before high-intensity training, it improves athletes’ time-trial performance and power outputs while reducing fatigue indices [16,22,46]. Although the present case did not involve limb immobilisation or direct assessment of muscle mass, prior studies indicate that IPC may attenuate muscle atrophy during immobilisation [47], with evidence that pairing IPC with neuromuscular electrical stimulation facilitates greater muscle preservation than IPC alone [48]. The passive nature of this approach enabled frequent use with minimal physiological strain, making it a low-energy adjunct useful for promoting recovery and supporting the metabolic and oxidative capacity required for competition in decathlons.

BFR aerobic cycling sessions were conducted to generate a meaningful cardiovascular response, serving as a proxy for high-intensity running sessions contraindicated by the injury [49]. Meta-analyses and controlled trials report that adding BFR to aerobic cycling yields greater improvements in VO2max, lower-limb strength, and muscle hypertrophy than matched low-intensity training without BFR, with the effects being comparable to traditional high-intensity cycling [50,51,52,53,54,55]. Mechanistically, limb occlusion increases local hypoxia and metabolic stress, augments anabolic signalling and fast-twitch fibre recruitment, and elicits transient endocrine responses, providing a plausible basis for concurrent aerobic and neuromuscular adaptations [53,55,56,57,58,59]. In this case, BFR cycling served as a timely method for maintaining cardiorespiratory fitness and lower-limb strength throughout rehabilitation without imposing high mechanical demands on the injured hamstring.

In contralateral strength training of the uninjured limbs, both with and without BFR, we leveraged the cross-education effect, wherein traditional high-intensity unilateral training induces contralateral strength adaptations, which are likely mediated by neural adaptations [60,61]. The addition of BFR to unilateral training may have enhanced adaptations through systemic physiological and hormonal responses [62,63,64]. Additionally, upper-body and aerobic BFR sessions may have supported systemic endocrine responses further promoting an anabolic and regenerative environment. Given the substantial modification of running, gym, and event-specific loads, the multimodal BFR program was a central adjunct intended to compensate for reduced mechanical stimuli. Although causality cannot be isolated in a single case, the athlete’s rapid restoration of function is consistent with reported effects of passive and active BFR interventions, which together may have contributed to maintaining readiness during this compressed timeline.

We employed a comprehensive, multimodal rehabilitation approach including physiotherapy, manual therapy, and conditioning alongside BFR training. Physiotherapy and manual therapy allowed for routine monitoring and soft-tissue management, while BFR provided the primary structured loading stimulus where traditional mechanical loading was restricted due to the injury. While this design precludes isolation of BFR’s specific contribution, it reflects real-world clinical practice, where interventions are rarely used in isolation. The use of 3–6 BFR sessions daily exceeded the usual conventional rehab session frequency (e.g., 2 sessions daily). The accelerated timeline observed in this case (with the athlete returning to full competition on day 18, which can be compared to the mean 18.6 days required for returning to full training) is noteworthy and provides preliminary evidence in support of high-frequency BFR; however, further research is warranted. Despite intensive programming (with up to six BFR-related sessions per day), continuous monitoring for skin irritation, numbness, or circulatory changes was maintained throughout, with no adverse events reported. The absence of adverse events further supports the safe application of multi-modal BFR under expert guidance. Collectively, the key factors contributing to the successful outcome included the athlete’s elite training status, the high level of competency in BFR application, and the effective implementation of key training principles such as individualisation and periodisation. Despite the ecological validity of this single-subject case report describing a highly motivated elite athlete who underwent a multimodal rehabilitation program, the limitations warrant mentioning. Specifically, without a comparator or control condition, the findings should be interpreted within the context of the athlete’s unique training history, competitive demands, and constrained competition timeline. Nevertheless, the data offers applied insight into how the application of high-frequency BFR may be pragmatically integrated when conventional loading is restricted.

## 5. Conclusions

This case report describes the application of a high-frequency BFR training program (3–6 sessions daily) to support return to elite competition on day 18 following a Grade 2b hamstring strain. The accelerated BFR rehabilitation framework suggests that BFR may provide both biological and mechanical mechanisms that promote muscular repair and preservation of neuromuscular function. While these outcomes highlight the potential utility of integrating high-frequency BFR within a multimodal rehabilitation framework, the findings reflect the experience of a single athlete. When applied under expert supervision, multimodal high-frequency BFR programming represents a viable strategy for accelerating functional restoration and preserving competitive readiness following an acute musculoskeletal injury.

## Figures and Tables

**Table 1:** Description of rehabilitation modalities prescribed during the accelerated rehabilitation program.

Modality	Description
Activation	Low-intensity lower-body preparatory exercises for subsequent sessions.
Upper-body BFR	Early-stage, band-resisted upper-body exercises wearing BFR cuffs used to generate systemic adaptive signalling while avoiding applying a mechanical load on the injured limb during the period of initial restricted lower-body training. Progressed to loaded resistance exercises later in Phase 1.
Right-leg strength BFR	Unilateral strength exercises for the uninjured leg conducted while the athlete was wearing BFR cuffs, serving as a high-load contralateral stimulus to maintain lower-limb strength and induce cross-education effects.
Lower-body strength	Higher-load compound movements (>70% 1 RM, e.g., trap bar deadlift and step-ups) reintroduced accordingly once the individual was pain-free.
Hamstring strengthening	Progressive isometric, eccentric, and isotonic hamstring-targeted exercises performed to restore strength and confidence.
Bike (stationary cycling)	Stationary cycling performed either as continuous steady-state (20 min @90RPM) or interval-based (8 × 15 s @130RPM/15 s @70RPM) cycling using a self-selected resistance setting with BFR applied to both thighs.
Track (running)	Running speed was self-limited by pain-free tolerance and athlete confidence. The distance was selected in consultation with the athlete according to what he felt he could achieve whilst maintaining good running mechanics.
Event-specific skills	Hurdles, high jump, long jump, pole vault, and throws, which were performed as modified or visualised movements to maintain technical familiarity.
Ischemic preconditioning	Performed in a seated position using three cycles of five minutes of inflation at 80% LOP, interspersed with three minutes of reperfusion using lower-body BFR cuffs. From Day 5, IPC was initially applied unilaterally (uninjured leg) and progressed to bilateral application.
Neuromuscular electro- stimulation	From Day 10, a second IPC session included low-frequency stimulation (Compex, DJO Global, Vista, CA, USA). Self-adhesive electrodes (5 × 5 cm) were positioned according to manufacturer guidelines: one electrode was placed over the distal portion of the hamstring muscle group (approximately two-thirds down the posterior thigh), and the second electrode was positioned proximally. Placement was adjusted to accommodate the position of the BFR cuff. Stimulation intensity was progressively increased until visible muscle contraction was observed without discomfort.

Abbreviations: BFR, Blood Flow Restriction; RM, repetition maximum; RPM, revolutions per minute; s, seconds; LOP, limb occlusion pressure; IPC, ischemic preconditioning.

**Table 2:** Summary of the accelerated rehabilitation program.

Day	Sessions	Programming	Running	Notes
Day 1	2 × day	UB-BFR band-resisted exercise	–	Initiation of rehabilitation; protection of injured left leg; early systemic activation using band-resisted upper-body BFR.
Day 2	3 × day	UB-BFR band-resisted exercise; R-leg STR; IPC	–	Right-leg BFR strength; left-leg glute/quad activation only; first IPC session—right-leg BFR only.
Day 3	4 × day	ACT; UB-BFR band-resisted exercise; TRACK; IPC	5 × 30 m	First run post-injury. Introduced SL Ham Iso-Push (three foot positions) to monitor force symmetry pre-run.
Day 4	6 × day	ACT; Bike; IPC; TRACK; IPC; Ham-STR	6 × 30 m	Bike (stationary cycling) without BFR. Left-hamstring-strength manual resistance.
Day 5	6 × day	ACT; TRACK; Bike; IPC; LB-STR; IPC	8 × 30 m	First high-load lower-body session. IPC—BFR on both legs.
Day 6	5 × day	ACT; Bike; IPC; TRACK; IPC	10 × 30 m	Bike (stationary cycling) BFR on both legs.
Day 7	5 × day	TRACK; Bike; IPC; LB-STR; Ham-STR; IPC	10 × 30 m	First gym-based hamstring specific strength session.
Day 8	6 × day	ACT; TRACK; IPC; Ham-STR, Bike; IPC + NMES	10 × 30 m	Addition of NMES to IPC in evening recovery session.
Day 9	5 × day	LB-STR; IPC; TRACK; Bike; IPC + NMES	Warm Up only	No run-specific rehab employed to allow for recovery.
Day 10	6 × day	ACT; IPC; TRACK; Ham-STR; Bike; IPC + NMES	6 × 30 m	Increased focus running speed.
Day 11	4 × day	LB-STR; IPC; TEST; IPC + NMES	–	Reduced volume for sprint test (day 12); running technique: athlete needed to display competency in LJ and HJ with short approach efforts.
Day 12	6 × day	ACT; IPC; TRACK; Ham-STR; Bike; IPC + NMES	60 m @95% vel	Sprint test. Passed flying 30 m and was cleared for competition.
Day 13	2 × day	TRACK; IPC + NMES	Warm Up only	Commenced taper; travel day.
Day 14	1 × day	IPC + NMES	–	Travel continued; no opportunity to train.
Day 15	4 × day	ACT; TRACK; Ham-STR; IPC + NMES	1000 m	Event specific tapering.
Day 16	3 × day	ACT; TRACK; IPC + NMES	5 × 60 m	Event specific tapering.
Day 17	3 × day	ACT; TRACK; IPC + NMES	Warm-up only	Final taper; mobility and activation.
Day 18	5 events	Olympic Games Competition	Competition	Day 1: 5 events completed. No performance in pole vault.
Day 19	5 events	Olympic Games Competition	Competition	Day 2: 5 events completed. Full decathlon completed without recurrence.

Abbreviations: BFR, Blood Flow Restriction; UB, upper-body; R-leg STR, right-leg strength; IPC, ischemic preconditioning; ACT, activation; Ham-STR, hamstring-specific strength; LB-STR, lower-body strength; NMES, neuromuscular electro-stimulation; m, metre; SL Ham Iso-Push, single-leg hamstring isometric push; LJ, long jump; HJ, high jump.

**Table 3:** Changes in eccentric hamstring strength (kg) throughout the rehabilitation program.

Day	Right (kg per Set)	Left (kg per Set)	Symmetry Index %
Day 7–8	32/39/39	25/25/25	64%
Day 10	32/39/39	32/32/32	82%
Day 12	32/39/39	32/39/39	100%

Symmetry Index % = (best left-leg load ÷ best right-leg load) × 100.

**Table 4:** Flying 30 m sprint performance during the rehabilitation phase.

Day	Sprint Time (s)	Relative Sprint Velocity %
Day 6	13.00	23%
Day 7	6.08	50%
Day 8	3.96	77%
Day 10	3.28	93%
Day 12	2.99	102%

Pre-injury time % was calculated as follows: (pre-injury reference time ÷ current time) × 100.

**Table 5:** Comparative performance data.

Event	Personal Best	Olympic Score	Difference
100 m (s)	10.77	10.98	+0.21
Long Jump (m)	7.62	7.36	–0.26
Shot Put (m)	13.24	13.35 *	+0.11 (PB)
High Jump (m)	2.11	2.05	–0.06
400 m (s)	47.84	49.02	–1.18
110 mH (s)	14.34	15.10	+0.76
Discus (m)	41.70	43.31 *	+1.61 (PB)
Pole Vault (m)	5.00	No height	-
Javelin (m)	62.48	58.52	–3.96
1500 m (min:s)	4:41	5:03	+0:22

* New personal best (PB).

## Data Availability

The data are available upon reasonable request.
